# Microglial Dynamics Modulate Vestibular Compensation in a Rodent Model of Vestibulopathy and Condition the Expression of Plasticity Mechanisms in the Deafferented Vestibular Nuclei

**DOI:** 10.3390/cells11172693

**Published:** 2022-08-29

**Authors:** Nada El Mahmoudi, Emna Marouane, Guillaume Rastoldo, David Pericat, Isabelle Watabe, Agnes Lapotre, Alain Tonetto, Christian Chabbert, Brahim Tighilet

**Affiliations:** 1 Aix-Marseille Université—Centre National de la Recherche Scientifique (CNRS), Laboratoire de Neurosciences Cognitives, LNC UMR 7291, Centre Saint-Charles, Case C, 3 Place Victor Hugo, CEDEX 03, 13331 Marseille, France; 2 Université de Caen (UNICAEN), INSERM, COMETE, Normandie Université, 14000 Caen, France; 3 Université de Toulouse Paul Sabatier-CNRS, Institut de Pharmacologie et de Biologie Structurale, 31400 Toulouse, France; 4 Aix Marseille Université—CNRS, Fédération Sciences Chimiques Marseille (FSCM) (FR 1739), PRATIM, Centrale Marseille, 13397 Marseille, France; 5 GDR Physiopathologie Vestibulaire Unité GDR2074, CNRS, 13003 Marseille, France

**Keywords:** microglia, inflammation, vestibular syndrome, vestibular nuclei, adaptative plasticity, neurogliogenesis, posturo-locomotor functions, vestibular compensation

## Abstract

Unilateral vestibular loss (UVL) induces a vestibular syndrome composed of posturo-locomotor, oculomotor, vegetative, and perceptivo-cognitive symptoms. With time, these functional deficits progressively disappear due to a phenomenon called vestibular compensation, known to be supported by the expression in the deafferented vestibular nuclei (VNs) of various adaptative plasticity mechanisms. UVL is known to induce a neuroinflammatory response within the VNs, thought to be caused by the structural alteration of primary vestibular afferents. The acute inflammatory response, expressed in the deafferented VNs was recently proven to be crucial for the expression of the endogenous plasticity supporting functional recovery. Neuroinflammation is supported by reactive microglial cells, known to have various phenotypes with adverse effects on brain tissue. Here, we used markers of pro-inflammatory and anti-inflammatory phenotypes of reactive microglia to study microglial dynamics following a unilateral vestibular neurectomy (UVN) in the adult rat. In addition, to highlight the role of acute inflammation in vestibular compensation and its underlying mechanisms, we enhanced the inflammatory state of the deafferented VNs using systemic injections of lipopolysaccharide (LPS) during the acute phase after a UVN. We observed that the UVN induced the expression of both M1 proinflammatory and M2 anti-inflammatory microglial phenotypes in the deafferented VNs. The acute LPS treatment exacerbated the inflammatory reaction and increased the M1 phenotype while decreasing M2 expression. These effects were associated with impaired postlesional plasticity in the deafferented VNs and exacerbated functional deficits. These results highlight the importance of a homeostatic inflammatory level in the expression of the adaptative plasticity mechanisms underlying vestibular compensation. Understanding the rules that govern neuroinflammation would provide therapeutic leads in neuropathologies associated with these processes.

## 1. Introduction

Unilateral vestibular loss (UVL) induces various deficits affecting posturo-locomotor, oculomotor, vegetative, and perceptivo-cognitive functions composing the so-called vestibular syndrome [1,2,3]. The origin of the vestibular syndrome was shown to be an electrophysiological asymmetry between the ipsi- and contra-lesional vestibular nuclei (VNs) [4,5,6], whose progressive disappearance over time supports the vestibular compensation, i.e., the spontaneous and progressive functional recovery after UVL [7]. Vestibular compensation is supported by the expression of several plasticity mechanisms in the ipsilesional VNs, such as the release of neurotrophic factors, changes in neuron membrane excitability, and reactional neurogliogenesis (for reviews, see [8,9]). UVL also induces an inflammatory response, witnessed by long-lasting astroglial and microglial reactions [10,11,12,13,14], and by the expression of the key inflammatory factors TNF-α and Nf-Kb in the ipsilesional VNs [15]. Inflammatory reaction within the VNs is observed in models of structural damage to the peripheral vestibular system [16] and is thought to be induced by the degeneration of vestibular nerve afferents, releasing inflammatory signals into the VNs. Furthermore, activation of the hypothalamo-pituitary-adrenal (HPA) axis has also been reported, leading to the release of endogenous anti-inflammatory corticosteroids (ECs) in the deafferented VNs [17,18], confirming UVN-induced neuroinflammatory process. We recently questioned the functional relevance of the acute inflammatory response after UVL using acute corticotherapy in a rat model of unilateral vestibular neurectomy (UVN) [11]. We observed that acute pharmacological blockade of the inflammatory response after UVN alters the expression of the plasticity mechanisms in the ipsilesional VNs and induces long-term deficits in the recovery of postural function. These results suggest a beneficial role for the acute inflammatory response in vestibular compensation and support clinical observations highlighting the poor benefits of acute corticotherapy in the management of vestibular patients [19,20,21]. The lack of benefits from anti-inflammatory compounds is not limited to the vestibular field since the same observations were made following traumatic brain injuries (TBI) both in humans [22,23,24,25] and animal models [26,27,28,29,30,31]. The classical view of detrimental neuroinflammation is increasingly being challenged and the idea that at least some inflammation is necessary for the brain’s self-repairing processes is emerging [25]. Indeed, the acute inflammatory response, as a self-regulated reaction, is thought to play a neuroprotective role by facilitating tissue repair and restoring the brain’s homeostasis [32]. However, the autoregulatory mechanism can be impaired leading to chronic inflammation, characterized by a self-propagating and long-lasting reaction leading to neurodegeneration and central nervous system (CNS) disorders [32,33,34]. The complexity of the inflammatory process is related to the complexity of the cellular actors involved. Inflammation involves microglial cells, astrocytes but also neurons, oligodendrocytes, endothelial cells, and tissue macrophages all participating in the inflammatory process [32,35,36]. Among all these actors, microglial cells appear to be a key component in the regulation of the inflammatory response, as they constitute the CNS’s first line of defense. Different states of microglial cells have been described: resting and activated [37,38]. In normal conditions, resting microglia continuously scan the environment to ensure brain homeostasis [39]. When danger is detected, microglia become ‘activated’ and release factors influencing surrounding cells’ functioning [38]. Among activated states, a broad spectrum has been described, going from the classical M1 pro-inflammatory, to the alternative M2 anti-inflammatory phenotypes [38,40,41]. M1 activation leads to the release of pro-inflammatory molecules and reactive oxygen species, to fight against the insult and clean the damaged area, while M2 activation leads to the release of anti-inflammatory molecules and promotes wound healing and recovery [37,38]. Sequential activation of both states would allow proper brain repair and resolution of the inflammation, but the maintenance of the M1 activation state could lead to chronic inflammation and neurotoxic effects [37]. Polarization of microglia has been well described in TBI [42] and spinal cord injury (for review see [43]), but to our knowledge, no study has reported microglial dynamics following UVL. Interestingly, systemic and cerebral injections of endotoxins such as lipopolysaccharide (LPS) have been shown to promote inflammation and M1 polarization [44,45]. While high doses of LPS are detrimental [46,47], low doses of LPS could be beneficial by causing immunostimulation and alleviating inflammatory response [48,49]. As the blockade of the inflammatory response after UVN was deleterious for both neuroplasticity mechanisms in the deafferented VNs and functional recovery, we wondered whether the slight stimulation of the inflammatory process with low doses of systemic LPS injections could be beneficial for vestibular compensation. In addition, in line with our previous study involving inhibition of the acute inflammatory response [11], stimulating the inflammatory process with LPS would enable us to understand its functional role following UVN. This study had two goals. Firstly, we aimed at characterizing microglial cells phenotypes dynamics during the inflammatory process induced by UVN. Secondly, we stimulated the acute inflammatory response after UVN with low doses of systemic LPS injections to observe its impact on the expression of plasticity markers in the ipsilesional VNs and on the kinetics of functional recovery.

## 2. Materials and Methods

### 2.1. Animals & Ethical Statements

This study was performed on 74 adult Long Evans female rats weighing between 250 and 350 g (10–12 weeks old at the beginning of the study). All experiments were performed under the European directive (2010/63/CE) which sets the regulations for the protection of animals used for scientific purposes and under the supervision of the veterinary and the National Ethical Committee (French Agriculture Ministry Authorization: 33363). Every attempt was made to minimize both the number and the suffering of animals used in this experiment. The animals were housed in a large, confined space with 12-h diurnal light variations with free access to water and food. They were housed at the Fédération 3C (Centre Saint-Charles, Aix-Marseille University) animal facility.

### 2.2. Study Design

**Figure 1:**
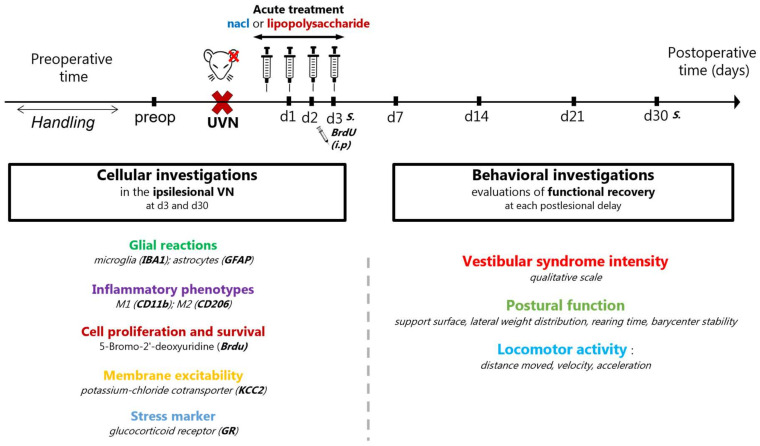
Study design. Experimental protocol used to study the effects of acute LPS injection following unilateral vestibular neurectomy (UVN) on vestibular compensation. Pharmacological treatments were administrated during the acute phase following UVN (first 3 days). Cellular investigations were performed in the ipsilateral vestibular nuclei (VN) at 3 and 30 days following the surgery. Behavioral investigations were used to evaluate the kinetics of vestibular compensation. A preoperative session was carried out to serve as a reference value for each rat. Then, evaluations were performed at 1, 2, 3, 7, 14, 21, and 30 postoperative days. S, sacrifice; i.p, intraperitoneal injections.

The study design (Figure 1) was complementary to our previous study aiming at understanding the impact of a blockage of the acute inflammatory response in vestibular compensation [11]. All experimental groups were part of the same protocol, aiming at understanding the pharmacological modulation of the acute inflammatory response during vestibular compensation. Animals were randomly trained at the same time, regardless of the experimental group, to avoid any bias. To understand the impact of an increased acute inflammatory response on vestibular compensation, we used a pro-inflammatory endotoxin (lipopolysaccharide -LPS-, 5 µg/kg, 2 mL/kg) administrated intraperitoneally (i.p) immediately after the UVN and during the acute phase (corresponding to the first 3 days after the lesion). We observed the effects of LPS treatment at both cellular and behavioral levels. Animals were handled during one week before the beginning of the experiments to ease up their stress during manipulations. Then they were randomly divided into 3 groups: a sham group (*n* = 27), submitted to the same surgical approach as UVN without sectioning the nerve; a UVN+placebo group (*n* = 22), lesioned and treated with placebo (NaCl 0.9%, 2 mL/kg); and a UVN+LPS group (*n* = 25), lesioned and treated with LPS. For cellular investigations, 4 animals from each group were sacrificed at the end of the acute phase (day 3) and 4 animals during the chronic phase (day 30), at the end of the behavioral study. At the cellular level, we looked for inflammatory and plasticity markers in the deafferented VNs at 3 and 30 days after the lesion. At the behavioral level, we measured the time course of vestibular compensation using different evaluations performed at different time points after the lesion (days 1, 2, 3, 7, 14, 21, and 30).

### 2.3. Unilateral Vestibular Neurectomy (UVN)

We used the model of left unilateral vestibular neurectomy (UVN) in the adult rat as previously described in [50]. Animals were anesthetized with isoflurane (4%) 30 min after a subcutaneous injection of buprenorphine (Buprecare^®^, Axiance, Pantin, France; 0.02 mg/kg). Animals were intubated, and the anesthesia was maintained during the surgery with isoflurane (3%). To access the left vestibular nerve, tympanic bulla approach was used (see [50] for details). The left vestibular nerve was sectioned at a post-ganglion level, close to the brainstem. For the sham group (*n* = 27), the surgery was limited to the perforation of the tympanic bulla. Before awakening, animals were injected subcutaneously with a solution of Ringer Lactate (Virbac, Carros, France; 10 mL/kg) to prevent dehydration resulting from the surgery. The success of the UVN (*n* = 47) was verified by the immediate appearance of a characteristic vestibular syndrome composed of postural, locomotor, and oculomotor symptoms [50].

### 2.4. Pharmacological Treatments

The pharmacological treatments were administrated once per day during the acute phase (the first three days after the UVN) [50]. The UVN+placebo group was administered NaCl 0.9% (2 mL/kg) while the UVN+LPS group was treated with lipopolysaccharide (LPS 5 µg/kg, 2 mL/kg), a pro-inflammatory endotoxin classically used to induce an inflammatory response in rodents. LPS was derived from *Escherichia coli O111:B4* serotype (Sigma-Aldrich, St. Louis, MO, USA) and stored at 4 °C until use. The dose was set to induce a systemic inflammatory response without interfering significantly with the animal’s posturo-locomotor behavior [51].

### 2.5. Cellular Investigations

#### 2.5.1. Tissue Preparation

Animals received an *i.p* injection of 5-Bromo-2’-deoxyuridine (BrdU: 200 mg/kg) dissolved in NaCl 0.9% 3 days after the lesion and were sacrificed either at 3 (*n* = 4 per group) or 30 days after the lesion (*n* = 4 per group) to assess, respectively, for cell proliferation and survival of the proliferative cells. Animals were deeply anesthetized with a mixture of ketamine (Imalgène 1000^®^, Merial, Lyon, France; 60 mg/kg) and medetomidine (Domitor^®^, Orion, Espoo, Finland; 0.25 mg/kg) for intracardiac perfusion. First, an injection of 400 mL of isotonic saline (0.9% NaCl) was performed, followed by an injection of 400 mL of freshly prepared solution (4% paraformaldehyde (PFA) and in 0.1 M phosphate buffer (PB), pH 7.4). Brains were then extracted and post-fixed overnight at 4 °C in PFA 4% solution. Brains were rinsed and cryoprotected by successive baths in sucrose solutions at increasing concentrations (10%, 20%, 30% of D-saccharose in 0.1M PB each for 24 h at 4 °C). Brains were then frozen in dry ice and cut into serial 40 µm frontal sections with a cryostat (Leica, Wetzlar, Germany) for immunochemistry.

#### 2.5.2. Immunohistochemistry

Immunohistochemistry was performed according to previously published protocols [11,12,52,53,54]. For BrdU immunohistochemistry, sections were incubated with a BrdU antibody (1:100, Dako, Santa Clara, CA, USA, M0744). For glucocorticoid receptor (GR) immunohistochemistry, we used a GR antibody (1:300, Thermo Fisher, Waltham, MA, USA, PA1-511A). For glial cells immunohistochemistry, we used a microglial marker, ionized calcium-binding adapter molecule 1 (IBA1) (1:2000, Wako, Richmond, VA, USA, Cat#019-19741), and an astrocyte marker, glial fibrillary acidic protein (GFAP) (1:200, Dako, Santa Clara, CA, USA, Z033401-2). We used CD11b (1:100, Santa Cruz, Dallas, TX, USA, SC-23937), also known as integrin αM, as a marker of the M1 proinflammatory phenotype of microglia [55,56]. Regarding the M2 phenotype, we used a marker for CD206 (1:500, RD System, Minneapolis, MN, USA, AF-2535), a mannose receptor expressed by M2 anti-inflammatory microglia [37,38]. For potassium–chloride cotransporter 2 (KCC2), sections were incubated with a KCC2 antibody (1:200, Merck, Darmstadt, Germany, 07-432). For each section, we used DAPI (1:5000, Merck, Darmstadt, Germany, D9542) to mark the nucleus. Sections were then incubated in secondary antibodies, a goat antirabbit conjugated with Alexa Fluor 488 (1:500, Invitrogen, Waltham, MA, USA, A11008) and a goat antimouse IgG conjugated with Alexa Fluor 594 (1:500, Invitrogen, Waltham, MA, USA, A11005). Finally, sections were mounted with Roti^®^Mount FluorCare medium (Roth, Warzawa, Poland, HP19.1).

#### 2.5.3. Cell-Counting Methods and Quantification of KCC2 Immunoreactivity

Cell counts were performed similarly to previously validated protocols [11,12,16,52,53]. All cellular investigations were performed in the left VNs at 3 and 30 days after left UVN or sham surgery. For quantification, 1 in 10 serial sections was used starting from the beginning (−9.84 mm relative to the bregma) to the end of the VNs (−13.08 mm relative to bregma [54]). Expression of the cellular markers was observed in the medial vestibular nuclei (MVN), and the lateral vestibular nuclei (LVN) for KCC2 as both nuclei are involved in posturo-locomotor functions. 15 ± 5 sections of the deafferented (left) VNs were assessed for each marker. Immunoreactive (ir) cells were analyzed using confocal imaging with a Zeiss LM 710 NLO laser scanning microscope equipped with a 63X/1.32 BA oil immersion lens. Numbers of CD11b-, CD206, IBA1-, GFAP-, GR-, and BrdU- immunoreactive (ir) cells were counted using an integrated microscopic counting chamber delineating the region of interest by a square of 425.10 mm^2^. For CD11b, CD206, and IBA1 immunostainings, a customized macro from Fiji software [55] was developed to process and analyze confocal images. The total number of marked structures for each signal channel and the number of colocalized structures were quantified for each image. For GR quantification, we calculated the percentage of GR staining colocalizing with the DAPI marker to assess GR nuclear localization reflecting its activation [56,57]. The quantification of the KCC2 immunolabeling was performed according to a previously published protocol [58] using a custom program written in Matlab^®^ (TheMathworks, Inc., Natick, MA, USA) allowing analysis of the fluorescence intensity at the plasma membrane of neurons (for details, see [11,58]). To avoid any bias, cellular analyses were performed under blind conditions.

### 2.6. Behavioral Investigations

For behavioral investigations, animals were randomly trained at the same time, regardless of the experimental group, to avoid any bias. For each test, investigations were performed before the surgery (preop) and at different delays (days) during the post-operative time (d1, d2, d3, d7, d14, d21, and d30) to evaluate the intensity of the vestibular syndrome and the time course of the vestibular compensation (Figure 1).

#### 2.6.1. Qualitative Evaluation of the Vestibular Syndrome

We assessed the intensity of the vestibular syndrome and its time course by using a cumulative qualitative scale listing typical deficits known to reflect the impairment of the vestibular function [50,59]. Each behavioral symptom corresponds to a score on the qualitative scale (tumbling: 5; retropulsion: 4; circling: 3; bobbing: 2; head-tilt: 1). The score corresponds to the sum of these different symptoms, reflecting the severity of the vestibular syndrome (see [50] for details). The effectiveness of the UVN was assessed at the awakening from surgery, by the immediate appearance of all symptoms.

#### 2.6.2. Support Surface Measurement after Tail-Lift Reflex (TLR)

Vestibular function is crucial for postural stability and posture-related responses [60]. We assessed the postural stability after UVN by measuring the support surface (i.e., the area between the four paws of the animal), a well-known indicator used in various models of vestibular loss [15,52,61,62]. To directly address the vestibular function, we performed the tail-lift reflex (TLR) [63] by holding the animal by the tail and subjecting it to vertical traction. Sudden vertical acceleration of the animal activates the remaining vestibular receptors while drastically reducing tactile and proprioceptive inputs [63,64,65,66]. Measurement of the support surface following TLR allows assessment of postural stability following vestibulospinal reflex and the indirect measurement of vestibular function’s recovery. Animals were placed in an open field and a picture was taken each time the animal landed after the TLR. The support surface was calculated in cm^2^, using an image analysis system developed on Matlab^®^(The Mathworks, Inc., Natick, MA, USA). For each animal, ten repeated measurements were taken and averaged before the operation (preop) and at each post-operative time-point starting from d3. For each rat, all measurements were normalized according to the preop value. TLR was not performed at d1 and d2 because of the severe posturo-locomotor deficits observed in animals at those time points.

#### 2.6.3. Assessment of Postural Function Using Dynamic Weight Bearing (DWB2^®^)

We used the second version of the dynamic weight-bearing device (DWB2^®^, Bioseb, Vitrolles, France) to quantify the postural function of rats under ecological conditions (for details see [61,66]). Animals were individually placed in the DWB2^®^ and moved freely for 5 min. The apparatus allows quantification of the support forces of each part of the animal’s body. We analyzed the weight distribution of the animals along the lateral axis, previously described as an indicator of unilateral vestibular deficit [61,66,67]. We measured the weight distribution between the right and left paws and calculated a laterality index for each rat, corresponding to the weight distributed on the right paws minus the weight distributed on the left paws. We also used the DWB2^®^ device to measure the rearing time (i.e., time spent on the two hind paws). It is considered an indicator of the animal’s ability to stand, reflecting its postural and balance control [66]. We also calculated posturographic parameters (barycenter position and dynamic during the acquisitions) at the pre-operative time point and two post-operative time points: d3, corresponding to the acute phase, and d30, corresponding to the chronic phase of the vestibular syndrome. Barycenter positions were quantified each time the animal was stationary and standing on its four paws. We assessed barycenter stability by measuring its maximum lateral deviation (Y_max deviation), and its inertia (measure of the dispersion of barycenter positions’ during the acquisitions). Both parameters reflect static postural stability (see [11]). For each rat, data were normalized according to pre-operative values.

#### 2.6.4. Assessment of Locomotor Activity in the Open Field

Locomotor activity was assessed by measuring different parameters affected by UVN in rats [11,68]. At the beginning of the session, rats were placed individually in the center of an open field (80 × 80 × 40 cm) for 10 min and tracked with Ethovision TM XT 14 software (Noldus, Wageningen, The Netherlands). We measured the total distance moved (cm), the mean velocity (cm/s), and the mean acceleration (cm/s^2^). Parameters were normalized for each rat according to the preoperative value.

### 2.7. Statistical Analysis

Statistical analyses were used to compare all three groups (sham, UVN+placebo, and UVN+LPS groups). Analyses were performed with GraphPad Prism 9 (GraphPad Software, San Diego, CA, USA). Data normality was controlled using the D’Agostino & Person normality test. For cellular data, two-way ANOVA was performed. For behavioral data, two-way repeated measure ANOVA was used. All tests were followed by post hoc Tukey’s multiple comparisons analysis. Results were considered significant at *p* < 0.05.

## 3. Results

### 3.1. Cellular Results

#### 3.1.1. Acute LPS Treatment Significantly Increases Glial Reactions in the Deafferented Medial Vestibular Nuclei (MVN)

Glial reactions are known to be crucial players of the inflammatory response in the CNS. To investigate the impact of acute LPS treatment on glial responses in the vestibular nuclei, we used 2 specific markers for microglia and astrocytes (respectively IBA1 and GFAP) and assessed their expression during the acute (d3) and chronic (d30) phases following UVN.

**Figure 2:**
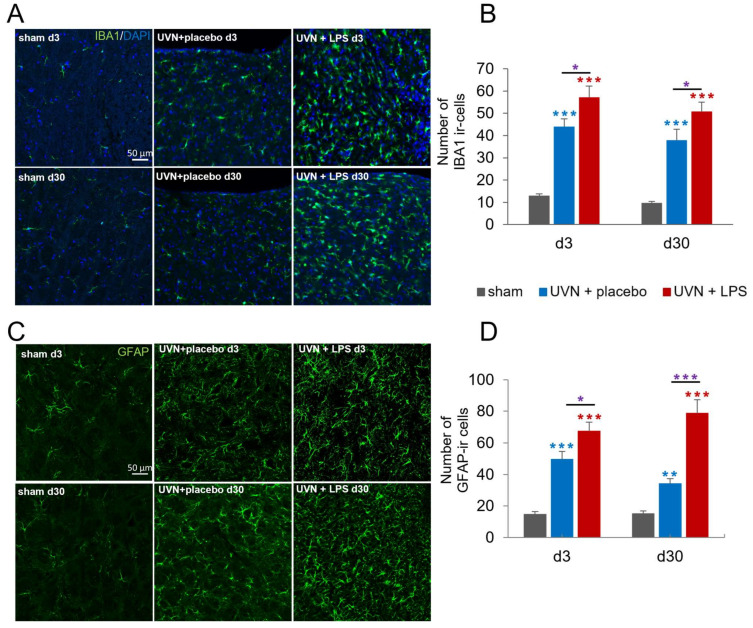
Glial reactions in the medial vestibular nuclei (MVN) are enhanced by the acute LPS treatment after UVN. (**A**) Confocal visualization of microglial cells immunostained with IBA1 and DAPI (nucleus) in the left MVN of a representative animal in sham, UVN+placebo, and UVN+LPS groups at d3 and d30. (**B**) Quantification of the number of IBA1 immunoreactive (ir) cells in the left MVN at d3 and d30. (**C**) Confocal visualization of astrocytes immunostained with GFAP in the left MVN of a representative animal in sham, UVN+placebo, and UVN+LPS groups at d3 and d30 post-UVN. (**D**) Quantification of the number of GFAP-ir cells in the left MVN at d3 and d30. Scale bar, 50 µm. Histograms represents the mean number of ir cells, with error bars representing SEM. * *p* < 0.05; ** *p* < 0.01; *** *p* < 0.001, two-way ANOVA, post hoc Tukey: sham and UVN+placebo comparison is indicated by a blue *; sham and UVN+LPS comparison is indicated by a red *; UVN+placebo and UVN+LPS comparison is indicated by a purple *.

The number of microglial cells was significantly increased following UVN and acute LPS treatment (Figure 2A,B). Statistical analysis revealed a significant ‘group’ effect (two-way ANOVA; ‘group’: F(2,191) = 76.36, *p* < 0.001). In the sham group, there was a stable number of IBA1-ir cells at d3 and d30. After UVN and placebo treatment, we observed a significantly increased number of IBA1-ir cells in the deafferented MVNs compared to the sham group at d3 (*p* < 0.001), persisting at d30 (*p* < 0.001). For the UVN+LPS group, there was a significant and persistent increase of IBA1-ir cells compared to both sham (*p* < 0.001) and UVN+placebo groups (*p* < 0.05) at d3 and d30.

Similarly, the number of GFAP-ir cells was significantly increased after UVN and acute LPS treatment (Figure 2C,D). The statistical analysis revealed significant ‘group’ and ‘group x time’ effects (two-way ANOVA; ‘group’: F(2,61) = 82.0, *p* < 0.001; ‘group x time’: F(2,61) = 4.26, *p* < 0.05). While an invariable number of GFAP-ir cells was observed over time in the sham group, there was a significant increase in the number of GFAP-ir cells in the deafferented MVNs in the UVN+placebo treatment compared to the sham group at d3 (*p* < 0.001) persisting at d30 (*p* < 0.01). Again, the UVN+LPS group, exhibited a significant and persistent increase of GFAP-ir cells compared to the sham group (*p* < 0.001) and the UVN+placebo group at both d3 (*p* < 0.05) and d30 (*p* < 0.001).

#### 3.1.2. M1 and M2 Expressions Are Induced in the Deafferented MVNs after UVN and Modulated by Acute LPS Treatment

To visualize microglial polarization and dynamics during the inflammatory response, we analyzed the expression of two markers used as specific markers of M1 (CD11b) [69,70] and M2 (CD206) [37,38] phenotypes combined with a classical marker of microglia (IBA1). Since these markers are not specific to reactive microglia and can be expressed by other cell types, we quantified the total number of immunoreactive(ir) cells, as well as the colocalization with the microglial marker IBA1.

**Figure 3:**
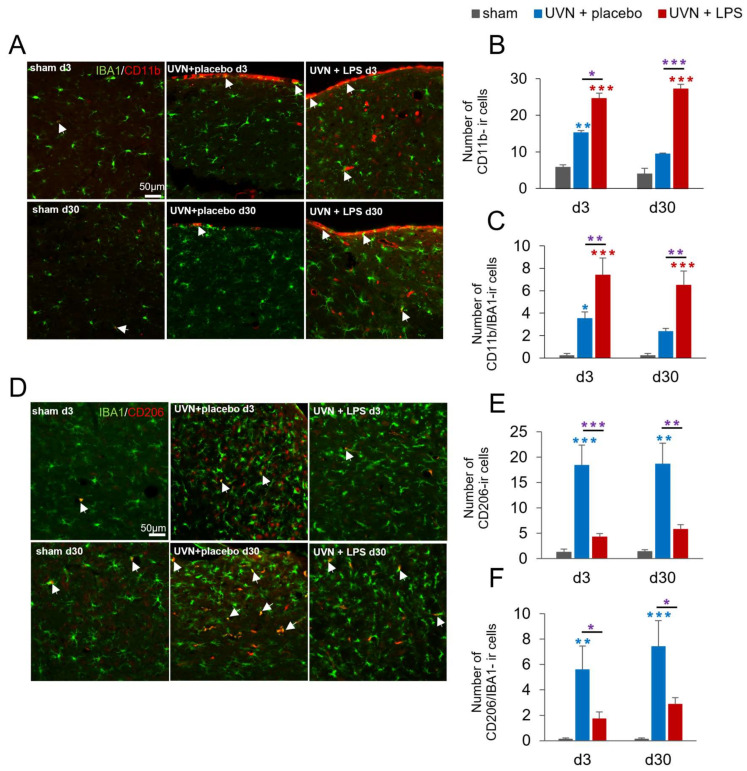
Expression of M1 proinflammatory and M2 anti-inflammatory phenotypes in the medial vestibular nuclei (MVN) are modified by the acute LPS treatment after UVN. (**A**) Confocal visualization of CD11b (M1 phenotype) and IBA1 (microglial cells) stainings in the left MVN of a representative animal in sham, UVN+placebo, and UVN+LPS groups at d3 and d30 post-UVN. Colocalization of both markers is represented by the merged color and examples are indicated by white arrows. (**B**) Quantification of the number of CD11b-immunoreactive (ir) cells in left MVN at d3 and d30. (**C**) Quantification of the number of CD11b/IBA1-ir cells in the left MVN at d3 and d30. (**D**) Confocal visualization of CD206 (M2 phenotype) and IBA1 (microglial cells) stainings in the left MVN of a representative animal in sham, UVN+placebo, and UVN+LPS groups at d3 and d30. Colocalization of both markers is represented by the merged color and examples are indicated by white arrows. (**E**) Quantification of the number of CD206-ir cells in the left MVN at d3 and d30. (**F**) Quantification of the number of CD206/IBA1-ir cells in left MVN at d3 and d30. Scale bar, 50 µm. Histograms represents the mean number of ir cells, with error bars representing SEM. * *p* < 0.05; ** *p* < 0.01; *** *p* < 0.001, two-way ANOVA, post hoc Tukey: sham and UVN+placebo comparison is indicated by a blue *; sham and UVN+LPS comparison is indicated by a red *; UVN+placebo and UVN+LPS comparison is indicated by a purple *.

For the M1 phenotype, CD11b- and IBA1/CD11b- ir cells were quantified (Figure 3A–C). Analyses revealed a significant increase in both quantifications after UVN and LPS treatment. Statistical analysis revealed significant ‘group’ effect for CD11b (two-way ANOVA; ‘group’: F(2,63) = 50.18, *p* < 0.001) and for CD11b/IBA1 colocalization (two-way ANOVA; ‘group’: F(2,54) = 27.96, *p* < 0.001). In the sham group, we observed a stable number of CD11b-ir cells over time with a very low colocalization with IBA1. In the UVN+placebo treatment, we observed a significant increase of CD11b-ir cells compared to the sham group at d3 (*p* < 0.01), disappearing at d30. Similarly, a significant proportion of IBA1/CD11b colocalization was observed in the UVN+placebo group compared to the sham group at d3 (*p* < 0.05) no longer present at d30. After UVN and LPS treatment, we observed a significant increase of CD11b-ir cells compared to the sham group (*p* < 0.001) and the UVN+placebo group (d3: *p* < 0.05; d30: *p* < 0.001). For this group, the number of IBA1/CD11b-ir cells was significantly increased compared to the sham group (*p* < 0.001) and to the UVN+placebo group (*p* < 0.01) at both d3 and d30.

For the M2 marker, CD206- and IBA1/CD206- ir cells were quantified (Figure 3D–F). We observed a significant decrease in both quantifications after UVN and LPS treatment. Statistical analysis revealed significant ‘group’ effect for CD206 (two-way ANOVA; ‘group’: F(2,65) = 19.11, *p* < 0.001) and for IBA1/CD206 colocalization (two-way ANOVA; ‘group’: F(2,81) = 16.02, *p* < 0.001). For the sham group, as for the M1 marker, we observed a stable number of CD206-ir cells in the MVN over time with almost no colocalization with IBA1. After UVN and placebo treatment, a significant increase of CD206-ir cells was observed in the deafferented MVN compared to the sham group at d3 (*p* < 0.001) still present at d30 (*p* < 0.01). The expression of IBA1/CD206-ir cells was also significantly enhanced compared to the sham group at d3 (*p* < 0.01) and d30 (*p* < 0.001). After UVN and LPS treatment, we observed no significant difference compared to the sham group in the number of CD206-ir cells while a significant decrease was observed at d3 (*p* < 0.001) and d30 (*p* < 0.01) compared to the UVN+placebo group. The proportion of IBA1/CD206-ir cells was not significantly different from the sham group while it was significantly reduced compared to the UVN+placebo group at d3 (*p* < 0.05) and d30 (*p* < 0.05).

#### 3.1.3. Acute LPS Treatment Significantly Increases KCC2 Expression in the Lateral VNs

The return to a physiological excitability level in the deafferented VNs is considered to be the neurobiological substrate of vestibular compensation [9]. A down regulation of the cation-chloride cotransporter KCC2 has been demonstrated at the level of the neuronal membrane in the deafferented vestibular neurons [52], conditioning a depolarizing action of GABA [71]. Our analysis was focused on the giant neurons of the lateral VNs as they contain excitatory glutamatergic neurons involved in vestibulospinal pathways. We used KCC2 expression to assess the impact of the acute LPS treatment on the neuron membrane excitability during both the acute (d3) and chronic phases (d30) following UVN.

**Figure 4:**
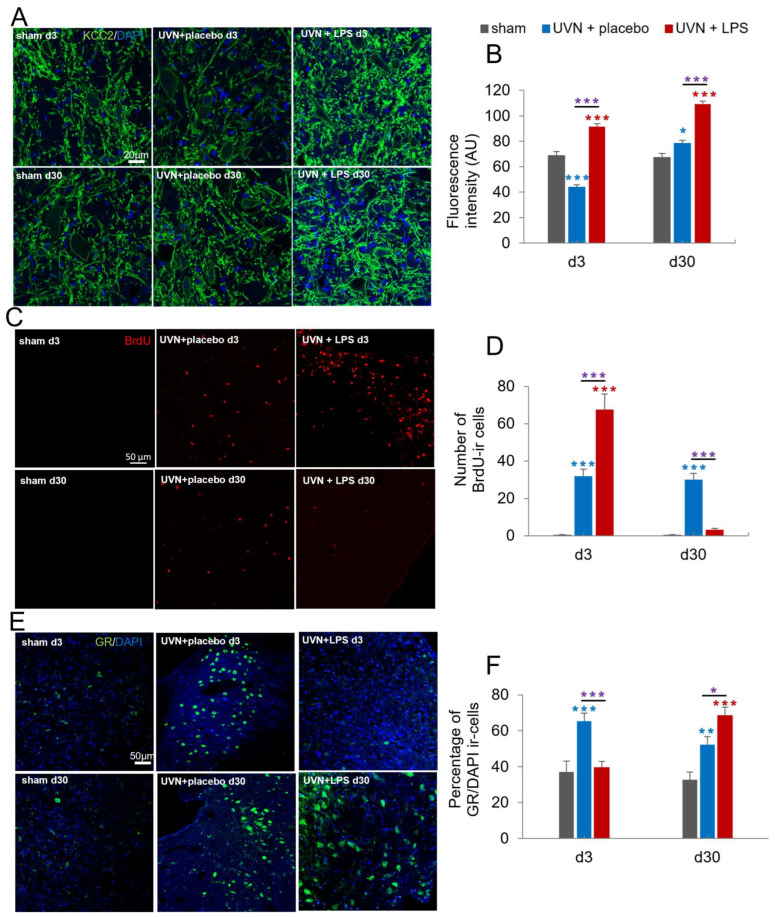
The expression of adaptative postlesional plasticity in the deafferented vestibular nuclei (VN) is altered following acute LPS treatment. (**A**) Confocal visualization of KCC2 and DAPI (nucleus) stainings in the left lateral vestibular nuclei (LVN) of a representative animal in sham, UVN+placebo, and UVN+LPS groups at d3 and d30. (**B**) Quantification of KCC2 fluorescence intensity at neuronal membrane in the left LVN at d3 and d30. (**C**) Confocal visualization of BrdU staining in the left medial vestibular nuclei (MVN) of a representative animal in sham, UVN+placebo, and UVN+LPS groups at d3 and d30. (**D**) Quantification of the number of BrdU-ir cells in the left MVN at d3 and d30. (**E**) Confocal visualization of glucocorticoid receptor (GR) and DAPI (nucleus) expression in the MVN of a representative animal in sham, UVN+placebo, and UVN+LPS groups at d3 and d30 post-UVN. (**F**) Quantification of the percentage of GR expression colocalizing with DAPI staining in the left MVN at d3 and d30. Scale bar, 50 µm. Histograms represents the mean number of ir cells, with error bars representing SEM. * *p* < 0.05; ** *p* < 0.01; *** *p* < 0.001, two-way ANOVA, post hoc Tukey: sham and UVN+placebo comparison is indicated by a blue *; sham and UVN+LPS comparison is indicated by a red *; UVN+placebo and UVN+LPS comparison is indicated by a purple *.

We observed a significant increase of KCC2 expression after UVN and LPS treatment (Figure 4A,B). Statistical analysis of KCC2 fluorescence intensity revealed significant ‘time’, ‘group’ and ‘group x time’ effects (two-way ANOVA; ‘time’: F(1,164) = 44.3, *p* < 0.001; ‘group’: F(2,164) = 77.4, *p* < 0.001; ‘time x group’: F(2,164) = 19.7, *p* < 0.001).

While KCC2 fluorescence intensity was constant over time for the sham group, it significantly decreased in the UVN+placebo group compared to the sham group at d3 (*p* < 0.001), and significantly increased 30 days after UVN (*p* < 0.05). On the contrary, in the UVN+LPS group, a significant increase in KCC2 fluorescence intensity was observed compared to the sham group (*p* < 0.001) and to the UVN+placebo group (*p* < 0.001) at both d3 and d30.

#### 3.1.4. Acute LPS Treatment Increases Cell Proliferation but Alters the Survival of New Cells in the Deafferented MVNs

Neurogliogenesis promoting vestibular compensation was revealed in deafferented VNs after UVN [12,53,72]. To evaluate the effect of acute LPS treatment on the rate of proliferation and cell survival in deafferented MVN at 3 and 30 days respectively, we used the BrdU marker (Figure 4C,D). Statistical analysis revealed significant ‘time’, ‘group’ and ‘group x time’ effects (two-way ANOVA; ‘time’: F(1,45) = 27.0, *p* < 0.001; ‘group’: F(2,45) = 23, *p* < 0.001; ‘time x group’: F(2,45) = 26, *p* < 0.001). In the sham group, we observed a very low number of BrdU-ir cells. In the UVN+placebo group, a significant increase in the number of BrdU-ir cells was observed in the deafferented MVN compared to the sham group at d3 (*p* < 0.001) and d30 (*p* < 0.001). In the UVN+LPS group, we observed a significant increase of BrdU-ir cells at d3 compared to the UVN+placebo group (*p* < 0.001) and the sham group (*p* < 0.001). However, at d30, we observed a significant decrease of BrdU-ir cells compared to the UVN+placebo group (*p* < 0.001), and no significant difference compared to the sham group.

#### 3.1.5. Acute LPS Treatment Significantly Alters GR Dynamics over Time in the Deafferented MVNs

Stress axis is activated after UVN [11,17,18] resulting in the release of endogenous corticosteroids activating glucocorticoid receptors (GR). GR is a cytosolic receptor, known to translocate into the nucleus once it is activated by a ligand [56,57]. To quantify the number of activated GR, we calculated the percentage of GR colocalizing with DAPI staining (nucleus) [11]. We quantified GR/DAPI colocalization to visualize the impact of acute LPS treatment on the stress response during both acute (d3) and chronic (d30) phases following UVN (Figure 4E,F). Statistical analysis revealed a significant ‘group’ effect (two-way ANOVA; ‘group’: F(2,81) = 16.02, *p* < 0.001). For the sham group, a basal and constant number of GR/DAPI ir-cells in the MVN was detected over time. After UVN and placebo treatment, a strong increase of GR/DAPI ir-cells was observed at d3 compared to the sham group (*p* < 0.001), still present at d30 (*p* < 0.01). In the UVN+LPS group, a significant decrease of GR/DAPI ir-cells was observed at d3 compared to the UVN+placebo group (*p* < 0.001) while no significant difference was found compared to the sham group. However, at d30, we observed a strong and significant increase in GR nuclear localization compared to both the UVN+placebo (*p* < 0.05) and sham (*p* < 0.001) groups.

### 3.2. Behavioral Results

#### 3.2.1. Acute LPS Treatment Significantly Increases Vestibular Syndrome Intensity during the Acute Phase

**Figure 5:**
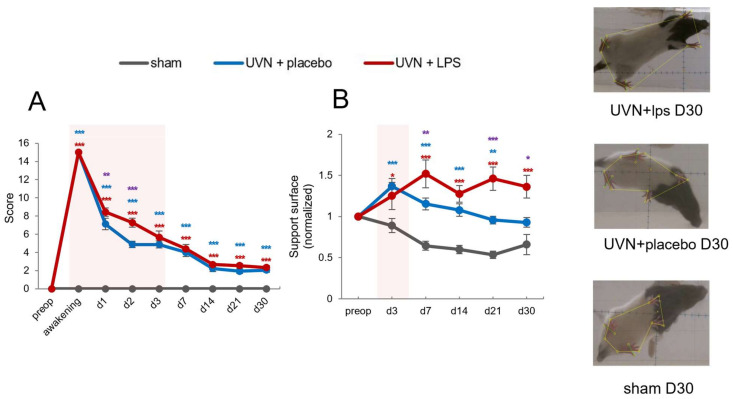
Acute LPS treatment enhances significantly vestibular syndrome intensity and postural instability following UVN. (**A**) Evolution of the score on the qualitative scale for the sham, UVN+placebo, and UVN+LPS groups. (**B**) Evolution of the support surface for the sham, UVN+placebo, and UVN+LPS groups. A light red box is positioned on the curve to indicate the period corresponding both to the acute phase of the vestibular syndrome (d1 to d3) and to the therapeutic window. Each point represents the group mean with error bars representing SEM. * *p* < 0.05; ** *p* < 0.01; *** *p* < 0.001, two-way RM ANOVA, post hoc Tukey: sham and UVN+placebo comparison is indicated by a blue *; sham and UVN+LPS comparison is indicated by a red *; UVN+placebo and UVN+LPS comparison is indicated by a purple *.

The posturo-locomotor component of UVN-induced vestibular syndrome was analyzed using a qualitative scale developed in our team [50].Vestibular syndrome intensity was significantly increased during the acute phase after UVN and LPS treatment (Figure 5A). Statistical analysis revealed significant ‘time’, ‘group’ and ‘time x group’’ effects (two-way repeated-measures ANOVA, ‘time’: F(8,392) = 354.2, *p* < 0.001; ‘group’: F(2,49) = 369.3, *p* < 0.001; ‘time x group’: F(16,392) = 89.76, *p* < 0.001). For the sham group, as expected, none of the deficits quantified by the qualitative scale were observed at any time points. A characteristic and previously reported pattern of posture-locomotor deficits was observed for the UVN+placebo group [11,50,61,66], with a severe syndrome at the awakening from surgery and during the acute phase after UVN, gradually decreasing over time (*p* < 0.001 compared to the sham group at all time points). In the UVN+LPS group, the score was significantly enhanced compared to the sham group (*p* < 0.001 at all-time points) and compared to the UVN+placebo group at d1 (*p* < 0.01) and d2 (*p* < 0.001), witnessing an increased vestibular syndrome intensity after acute LPS treatment during the acute phase.

#### 3.2.2. Acute LPS Treatment Significantly Increases Support Surface Measured after Vestibulospinal Reflex

Postural stability and effectiveness of the vestibulospinal reflex following tail-lift reflex (TLR) were analyzed by measuring the support surface [63,64,65,66]. An increase of the support surface is observed during the vestibular syndrome in different animal models [15,61,73] and is thought to reflect postural instability following unilateral vestibular loss (UVL). We observed a significant increase in the support surface after UVN and LPS treatment (Figure 5B). Statistical analysis revealed significant ‘time’, ‘group’ and ‘time x group’ effects (two-way repeated-measures ANOVA, ‘time’: F(5,130) = 4.65, *p* < 0.001; ‘group’: F(2,26) = 18.7, *p* < 0.001; ‘time x group’: F(10,130) = 8.68, *p* < 0.001). As previously described [11], the sham group presented a reduction of the support surface over time attesting an habituation to the test. A typical expression pattern for the support surface was observed in the UVN+placebo group with a significant increase from d3 (*p* < 0.001) to d21 (*p* < 0.01) compared to the sham group. For the UVN+LPS group, a strong and significant increased support surface was observed compared to both sham (*p* < 0.001) and UVN+placebo groups (from d3: *p* < 0.01 to d30: *p* < 0.05), highlighting enhanced and prolonged alteration of both postural stability and vestibulospinal reflex over time for the UVN+LPS group.

#### 3.2.3. Effect of Acute LPS Treatment on Postural Function

##### Weight Distribution along the Lateral Axis

**Figure 6:**
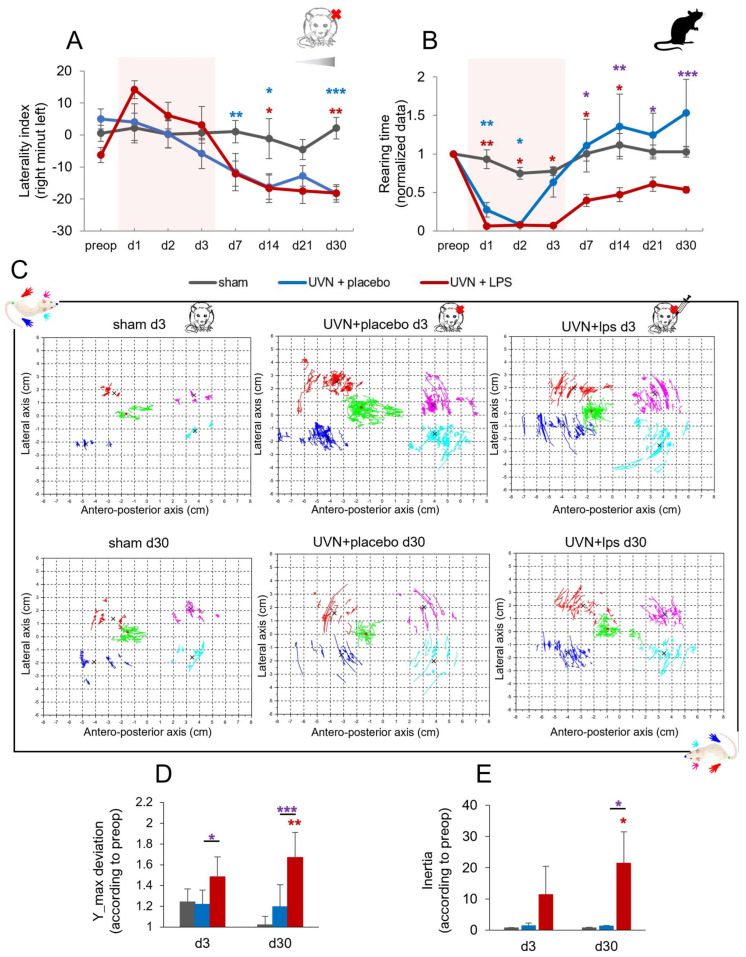
Postural function is significantly altered following acute LPS. (**A**) Evolution of the laterality index. Laterality index represents the lateral weight distribution and is calculated as follows: weight distributed on the right paws minus weight distributed on the left paws. (**B**) Evolution of the rearing time. Data are normalized according to the preoperative value. A light red box is applied on the curve to illustrate the acute time window of the vestibular syndrome (d1 to d3) where the treatments were administrated daily. (**C**) Statokinesiograms of a representative animal of sham, UVN+placebo and UVN+LPS groups at d3 and d30. Green clusters correspond to the positions of the barycenter during an acquisition, when the animal was on its four paws and stationery. It reflects postural stability in static conditions. The red, blue, magenta, and cyan dot clouds correspond, respectively, to the left and right hind paws and the left and right forepaws. (**D**) Quantification of the barycenter’s maximum lateral deviation. Data are normalized according to the preoperative value. (**E**) Quantification of the barycenter’s inertia, reflecting the dispersion of the barycenter’s positions. Data are normalized according to the preoperative value. Each data point and histogram represent the group mean with error bars representing SEM. * *p* < 0.05; ** *p* < 0.01; *** *p* < 0.001, two-way RM ANOVA, post hoc Tukey: sham and UVN+placebo comparison is indicated by a blue *; sham and UVN+LPS comparison is indicated by a red *; UVN+placebo and UVN+LPS comparison is indicated by a purple *.

Weight distribution on the lateral axis is a good index of postural stability [61,66,67,74] and was considered as a laterality index corresponding to the weight distributed on the right paws minus the weight distributed on the left paws (Figure 6A). A significant increase in the weight distributed on the left paws was observed during the chronic phase of vestibular compensation. For both UVN+placebo and UVN+LPS groups. Statistical analysis revealed significant ‘time’, ‘group’ and ‘time x group’’ effects (two-way repeated-measures ANOVA, ‘time’: F(7,140) = 12.2, *p* < 0.001; ‘group’: F(2,20) = 4.36, *p* < 0.05; ‘time x group’: F(14,140) = 3.42, *p* < 0.001). Lateral weight distribution was homogenous for the sham group and stable over time. As previously described [11,61,66,67,74], a significant diminution of the laterality index (witnessing an increase of the weight distribution towards the left paws) was observed in the UVN+placebo group compared to the sham group from d7 (*p* < 0.01), still present at d30 (*p* < 0.001). For the UVN+LPS group, the same pattern was observed with a significant decrease of the laterality index at d14 (*p* < 0.05), still present at d30 (*p* < 0.01). No significant difference was observed between the UVN+LPS and UVN+placebo groups.

##### Rearing Time

Rearing time is a good indicator of static postural control as it reflects the animals’ ability to stand [66]. We analyzed the evolution of the rearing time (Figure 6B) and observed a significant decrease after UVN and LPS treatment. Statistical analysis revealed significant ‘time’, ‘group’ and ‘time x group’ effects (two-way repeated-measures ANOVA, ‘time’: F(7,140) = 14.42, *p* < 0.001; ‘group’: F(2,20) = 6.122, *p* < 0.001; ‘time x group’: F(14,140) = 3.6, *p* < 0.001). In the sham group, the rearing time is stable over time. After UVN and placebo treatment, a significant diminution of the rearing time was observed compared to the sham group at d1 (*p* < 0.01) and d2 (*p* < 0.05). For the UVN+LPS group, a significant diminution of the rearing time is observed compared to the sham group at d1 (*p* < 0.01) persisting until d14 (*p* < 0.05). Compared to the UVN+placebo group, a significant diminution of the rearing time is observed in the UVN+LPS group at d7 (*p* < 0.05), still present at d30 (*p* < 0.001) reflecting an impairment of postural control recovery.

##### Barycenter Posturographic Analysis

An analysis of the barycenter has been used to assess the static equilibration function similarly to posturographic evaluations performed in patients with vestibular disorders [61,75,76]. Statokinesiograms were plotted for each animal, representing barycenter and paw positions calculated each time the animal was motionless and standing on its four paws (Figure 6C). Although illustrative, we can observe on the statokinesiograms a greater dispersion of the green dots (representing the barycenter positions) during the acute phase for the UVN+placebo and UVN+LPS groups. During the chronic phase, the dispersion for the UVN+placebo seems slighter while it persists in the UVN+LPS group. To quantify quantitatively the stability of the barycenter we used 2 parameters: the maximum lateral deviation and the inertia of the barycenter (for details see [11]).

##### Barycenter Maximum Lateral Deviation

The stability of the barycenter along the lateral axis was calculated with the barycenter maximum lateral deviation (Figure 6D). Statistical analysis revealed significant ‘group’ effect (two-way repeated-measures ANOVA, ‘group’: F(2,20) = 7.026, *p* < 0.01). We observed a significant increase in this parameter in the UVN+LPS group compared to the UVN+placebo group (d3: *p* < 0.05; d30: *p* < 0.001) and to the sham group (d30: *p* < 0.001) reflecting a significant and persistent increase of barycenter lateral instability for this group.

##### Barycenter Inertia

Rodents’ stability was assessed by measuring barycenter inertia which is the dispersion of barycenter positions during the acquisitions (Figure 6E). Statistical analysis revealed significant ‘time’ and ‘group x time’ effects (two-way repeated-measures ANOVA, ‘time’: F(1,21) = 16.97, *p* < 0.001; ‘group x time’: F(2,21) = 17.85, *p* < 0.001). We observed a significantly increased inertia in the UVN+LPS group at d30, compared to both sham (*p* < 0.05) and UVN+placebo groups (*p* < 0.05) witnessing a more pronounced barycenter instability.

#### 3.2.4. Effect of Acute LPS Treatment on Locomotion

UVN is known to induce locomotor deficits during the acute phase followed by a characteristic locomotor phenotype during the compensated phase [68]. Here we analyzed the impact of the acute LPS treatment on locomotor parameters known to be affected by UVN.

##### Total Distance Traveled

**Figure 7:**
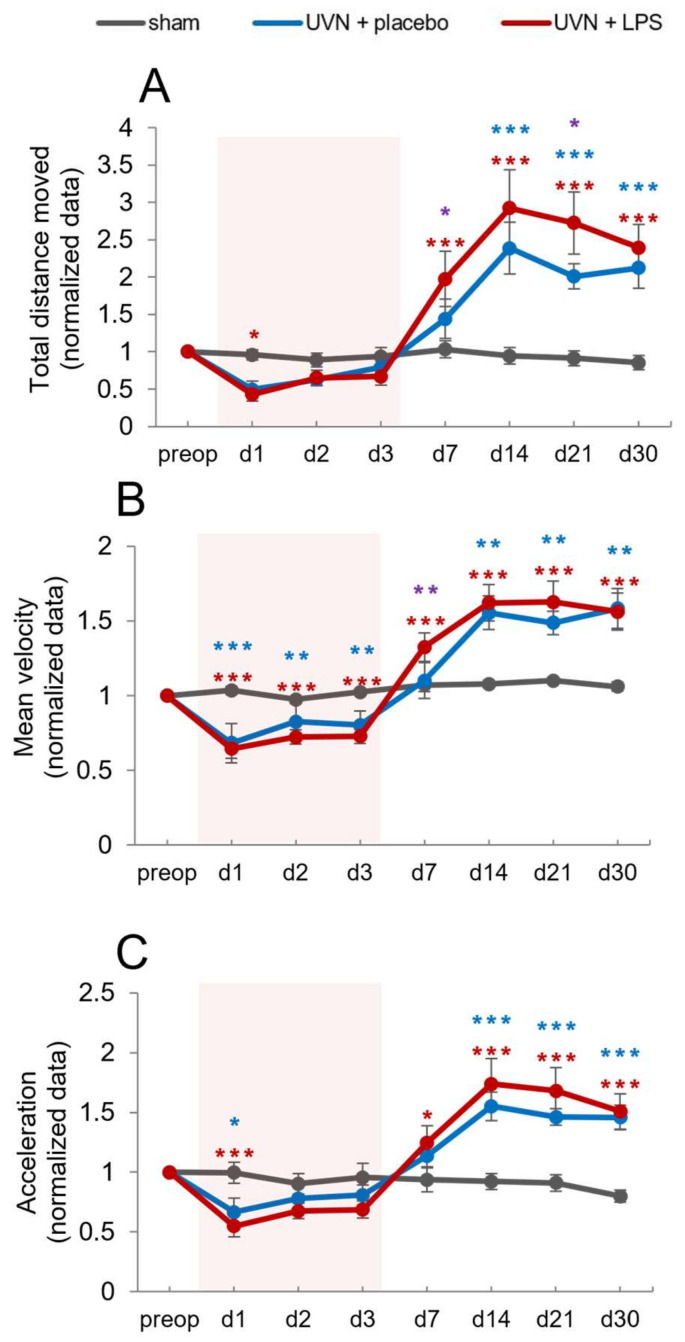
Acute LPS treatment has no impact on locomotor activity following UVN. (**A**) Evolution of the total distance moved. (**B**) Evolution of the mean velocity. (**C**) Evolution of the acceleration. Data are normalized according to preop values. A light red box is positioned on the curve to indicate the period corresponding to both the acute phase of the vestibular syndrome (d1 to d3) and the therapeutic window. Each data point represents the group mean with error bars representing SEM. * *p* < 0.05; ** *p* < 0.01; *** *p* < 0.001, two-way RM ANOVA, post hoc Tukey: sham and UVN+placebo comparison is indicated by a blue *; sham and UVN+LPS comparison is indicated by a red *; UVN+placebo and UVN+LPS comparison is indicated by a purple *.

Total distance traveled in the open field was quantified during the acquisitions. We observed a significant increase in this parameter after UVN for both UVN+placebo and UVN+LPS groups in the chronic phase (Figure 7A). Statistical analysis revealed significant ‘time’, ‘group’ and ‘time x group’ effects (two-way repeated-measures ANOVA, ‘time’: F(7,224) = 58.99, *p* < 0.001; ‘group’: F(2,32) = 10.14, *p* < 0.001; ‘time x group’: F(14,224) = 24.11, *p* < 0.001). As already described [11,68], we observed a significant increase in the total distance traveled in the UVN+placebo group, compared to the sham group at d14 (*p* < 0.001) still present at d30 (*p* < 0.001). For the UVN+LPS group, we observed a significant increase compared to the sham group at d7 (*p* < 0.001) still present at d30 (*p* < 0.001). When comparing the UVN+placebo and UVN+LPS groups, we observed overall the same pattern although a significant increase of this parameter was observed at d7 (*p* < 0.05) and d21 (*p* < 0.05).

##### Mean Velocity

For the mean velocity, we observed a similar time course after UVN for both UVN+placebo and UVN+LPS groups (Figure 7B). Statistical analysis revealed significant ‘time’ and ‘time x group’ effects (two-way repeated-measures ANOVA, ‘time’: F(7,224) = 119, *p* < 0.001; ‘time x group’: F(14,224) = 32.7, *p* < 0.001). As previously described [11,68], a significant decrease in mean velocity was observed in the UVN+placebo group compared to the sham group from d1 (*p* < 0.001) to d3 (*p* < 0.01). Conversely, a significant opposite effect was observed from d14 (*p* < 0.01), persisting at d30 (*p* < 0.001). For the UVN+LPS group, a similar pattern was observed compared to the sham group (*p* < 0.001 at all-time points). Compared to the UVN+placebo group, a similar pattern was observed except for a significant increase in the mean velocity at d7 (*p* < 0.01).

##### Mean Acceleration

Quantitative assessment of animal accelerations in motion is a way to account for vestibular dysfunction after UVN [68]. We observed a similar time course of mean acceleration for both UVN+placebo and UVN+LPS groups (Figure 7C). Statistical analysis revealed significant ‘time’ and ‘time x group’ effects (two-way repeated-measures ANOVA, ‘time’: F(7,224) = 42.22, *p* < 0.001; ‘time x group’: F(14,224) = 19.81, *p* < 0.001). As previously described [11,68], a significant decrease in the mean acceleration was observed in the UVN+placebo group compared to the sham group at d1 (*p* < 0.05) followed by a significant increase at d14 (*p* < 0.001) still present at d30 (*p* < 0.001). For the UVN+LPS group, the same pattern was observed compared to the sham group (d1: *p* < 0.001; d30: *p* < 0.001). No significant difference between the UVN+placebo and UVN+LPS groups was observed.

## 4. Discussion

Microglial cells are a key component of brain inflammation, involved both in the onset and the resolution of the inflammatory response [32,37,77]. It is now known that Unilateral Vestibular Loss (UVL) induces a long-lasting microglial reaction in the deafferented vestibular nuclei (VNs) [10,11,12,14,74]. We recently demonstrated that the pharmacological blockade of the acute inflammatory response after UVN inhibits microglial reaction and alters the expression of the endogenous plasticity mechanisms in the deafferented VNs. Furthermore, these alterations are associated with enhanced and persistent functional deficits [11]. Although these results suggest a beneficial role for acute endogenous inflammation and microglial reaction in vestibular compensation, to our knowledge nothing is known about M1/M2 microglial dynamics following UVL. Here, we studied the expression of two specific markers of M1 pro-inflammatory (CD11b) and M2 anti-inflammatory (CD206) phenotypes during both acute and chronic phases of vestibular compensation in the rodent UVN model. We demonstrated here a concomitant expression of both M1 and M2 phenotypes in the deafferented VN with different kinetics following UVN. Furthermore, the dynamics of M1 and M2 phenotypes are temporally correlated with the expression of endogenous plasticity mechanisms in deafferented VNs as well as with functional recovery. After acute LPS pharmacological treatment, we observed a strong up-regulation of the M1 phenotype, associated with altered plasticity mechanisms in the deafferented VN and enhanced postural deficits. Our results highlight, for the first time, the crucial role of M1 pro-inflammatory and M2 anti-inflammatory dynamics in vestibular compensation and open the way for new therapeutic procedures in vestibular patients with poor functional recovery.

### 4.1. Microglial Dynamics following UVN and Impact of the Acute LPS-Treatment

Microglia are highly plastic cells, constituting the CNS’s first line of defense. The microglial reaction has been classically studied using the IBA1 marker, a calcium-binding protein, whose expression is strongly up-regulated during inflammation and microglial activation in the brain [38,78]. In accordance with previous works, we observed a strong and long-lasting increase of IBA1-ir cells in the deafferented VNs following UVN [10,11,12,14,52,74]. Following acute LPS treatment, the microglial reaction was exacerbated during both the acute and the chronic phases following UVN suggesting an enhanced and persistent inflammatory response and microglial activation.

Traditionally, activated microglia are classified as being either M1 pro-inflammatory or M2 anti-inflammatory and M1 and M2 appear to be the limits of a broad spectrum of phenotypes extending from the pro-inflammatory and potentially cytotoxic M1 to the anti-inflammatory and neuroprotective M2 [38]. However, this binary classification is question of debate [79] and have been challenged for a few years. Indeed, M1 and M2 classification is based mainly on in vitro studies of peripheral macrophages while brain microglia appear to express a mixed phenotype of both states instead [80,81]. That being said, the use of M1 and M2 markers of microglia is, nevertheless, a useful tool to discriminate between microglial function in the brain microenvironment and the balance of M1/M2 microglial markers have been used and implicated in several neurodegenerative diseases [41].

Here, we used CD11b, also known as integrin αM, as a marker of M1 microglia [69,70]. CD11b forms a heterodimeric integrin (αMβ2) also known as macrophage-1-antigen (Mac-1). Interestingly, the production of the key pro-inflammatory cytokine TNF-α has been shown to colocalize with the expression of Mac-1 in microglial cells [82] supporting the idea that CD11b is associated with a pro-inflammatory function of microglia. Moreover, MAC-1 is upregulated in neurodegenerative diseases suggesting that it could be associated with a neurotoxic function of microglia [83]. Regarding the M2 anti-inflammatory function of microglia, we used the classical marker CD206 [37,38] also known as the mannose receptor, which is a receptor localized in cellular and endosomal membranes, involved in phagocytosis but most importantly in the resolution of the inflammatory process [84]. CD206 expression has indeed been shown to be increased during the resolution of the inflammation [85], making this marker appropriate for the M2 anti-inflammatory function of microglia.

Here, we observed the co-existence of both M1 and M2 markers in the deafferented VNs during the acute phase after UVN. While M1 expression decreases over time, we observed a long-lasting M2 expression. The M1 kinetics observed in our UVN rodent model is in line with previous work reporting an increased expression of the pro-inflammatory factors NF-kB and TNF-α in the deafferented VNs during the acute phase following UVL, progressively disappearing over time [15]. Interestingly, the authors also reported a delayed and long-lasting upregulation of the manganese superoxide dismutase (MnSOD), an enzyme involved in neuroprotection and neuronal survival [86,87]. These observations suggest the presence of a pro-inflammatory environment in the deafferented VN during the acute phase following UVL progressively disappearing over time while an anti-inflammatory environment occurs in parallel, with a long-lasting expression of molecules involved in neuroprotection and the resolution of the inflammatory process.

Following acute LPS-treatment, we observed a massive and long-lasting increase of the M1 pro-inflammatory expression with significant inhibition of the M2 anti-inflammatory phenotype during both the acute and chronic phases following UVN. LPS is recognized by pattern recognition receptors (PRRs), expressed widely on mammalian immune cells. In particular, LPS binds to the cluster of differentiation 14 (CD14), which forms a multi-receptor complex along with the toll-like receptor 4 (TLR4) and the Myeloid Differentiation factor 2 (MD-2), leading to the activation of the transcriptional factor Nf-Kb and the release of pro-inflammatory cytokines [88]. In our study, LPS injections were performed intraperitoneally, which is known to increase levels of pro-inflammatory cytokines both in serum [51] and in the CNS [89]. The question of whether peripheral injection of LPS could infiltrate the brain is still a matter of debate. While some studies reported that the LPS doesn’t cross the blood-brain barrier [90], others observed infiltration of peripheral LPS into the CNS [91,92]. Nevertheless, systemic inflammation caused by peripheral injection of LPS induces microglial reaction within the CNS through the activation of the NF-KB pathway, leading to the release of pro-inflammatory cytokines [93] which may explain the significant increase of microglial reaction and the massive M1 phenotype expression in our UVN model following acute LPS-treatment.

Surprisingly, among all CD11b and CD206 immunoreactive cells labeled in our study, only a small proportion colocalize with IBA1. Although IBA1 up-regulation is associated with microglial activation, it should be noted that IBA1 is not specific to reactive microglia as it can also be used as a marker of resting-state microglia [38,94]. In addition, CD11b and CD206 are also known to be expressed by other cell types such as peripheral immune cells [38,95], tissue macrophages [35], and astrocytes [96] which are also part of the inflammatory process. The lack of specificity of these markers for reactive M1 and M2 microglia could explain why only a small proportion of IBA1/CD11b and IBA1/CD206 were observed in our study. Nevertheless, considering the functional role of CD11b and CD206 during brain inflammation discussed above, it seems reasonably correct to consider that CD11b expression is associated with a pro-inflammatory function and CD206 with an anti-inflammatory function, whatever the cell type expressing the marker. Furthermore, despite the lack of specificity for reactive microglia of the markers used here, we observed that the acute LPS treatment in our UVN model favors CD11b ir-microglia expression over CD206-ir microglia, which is in accordance with the classical LPS action promoting macrophage and microglial polarization toward the M1 pro-inflammatory phenotype [77].

### 4.2. M1 and M2 Phenotypes as Regulators of the Plasticity in the Deafferented VN

#### 4.2.1. Reactive Astrocytes Are Regulated by the Inflammatory State of the Deafferented VNs

Like microglia, astrocytes are involved in the CNS inflammatory response [32,97]. Classically, astrocyte activation during inflammation is witnessed by the GFAP protein, an intermediate filament specific to astrocytes, which has been shown to increase during CNS insults [98]. Here, reproducing previous works, we observed a strong and long-lasting astrocyte reaction in the deafferented VNs [11,12,13,16]. After acute LPS treatment, astrocyte reaction was significantly enhanced during both the acute and the chronic phases following UVN, suggesting a modulation of astrocyte reaction by the inflammatory state of the environment. We indeed observed the disappearance of the astrocyte reaction under acute anti-inflammatory treatment [11]. In particular, reactive microglial cells are known to beactivated first under pathological conditions, and to initiate astrocyte reaction [99]. Since the microglial reaction was enhanced following acute LPS treatment, it is therefore likely that it caused an enhanced astrocyte reaction. Interestingly, it was shown that, through the release of pro- and anti-inflammatory cytokines, reactive microglia can condition reactive astrocytes into both neurotoxic [40] and neuroprotective [100,101] phenotypes, referred to respectively as A1 and A2 [102]. We could argue that microglial polarization in our UVN model conditions astrocytes’ phenotypes. While both A1 and A2 phenotypes might be present after UVN, acute LPS treatment could potentially promote astrocytes’ polarization towards a neurotoxic phenotype A1 thus contributing to the disruption of both plasticity mechanisms in the deafferented VNs and functional recovery.

#### 4.2.2. Neurogliogenesis Is Modulated by M1/M2 Polarization in the Deafferented VNs

Reactive neurogliogenesis has been described in the deafferented VN as a key parameter for vestibular compensation following UVN [12,53]. Traditionally, neurogliogenesis is divided into several steps including cell proliferation, survival, and differentiation [103]. We recently showed that the inhibition of the acute inflammatory response following UVN altered both cell proliferation and survival in the deafferented VN [11], suggesting that neurogliogenesis after UVN is somehow dependent on the acute inflammatory process. Following acute LPS treatment, we observed a strong and significant increase in cell proliferation during the acute phase but almost no survival of the proliferative cells during the chronic phase following UVN. Based on these observations, cell proliferation during the acute phase could be governed by the M1 pro-inflammatory state of the environment while the survival of the proliferative cells could depend on an M2 anti-inflammatory environment. Pro-inflammatory cytokines released by pro-inflammatory microglia such as TNF-α and IL-1β are indeed known to stimulate cell proliferation [104,105]. However, they alter neurogliogenesis by having detrimental effects on the survival and differentiation of newborn cells [104,106,107]. In contrast, M2 anti-inflammatory microglia promote neurogliogenesis [106,108] through the release of anti-inflammatory cytokines and neurotrophic factors playing a supportive role in the survival and differentiation of newborn cells [107]. Interestingly, LPS injection in the brain, by promoting M1 microglia, was shown to have detrimental effects on neurogenesis that were reversed by the administration of minocycline [109], known to inhibit M1 microglial polarization and promote M2 anti-inflammatory microglia [70,110,111]. Therefore, since they act in different steps, sequential expression of both M1 and M2 phenotypes seems necessary for the expression of adaptative neurogliogenesis in the deafferented VNs.

#### 4.2.3. KCC2 Expression Is Modulated by the Inflammatory Response following UVN

We previously examined the state of neuronal excitability of the deafferented vestibular neurons using the expression of the cation-chloride cotransporter KCC2 (which determines the hyperpolarizing action of GABA). KCC2 is downregulated in the deafferented VN during the acute phase following UVN [52]. This process may induce an intracellular accumulation of [Cl-] ions, leading to a depolarizing action of GABAA receptors [52,71,112]. As the key parameter for vestibular compensation is the restoration of the spontaneous activity in the deafferented VN [8,113,114], this transient mechanism seems to be a compensatory strategy, capable of re-establishing a level of homeostatic excitability in the ipsilesional VNs. Following acute LPS-treatment, we observed a significant increase in KCC2 expression during both the acute and chronic phases following UVN, potentially enhancing neuronal inhibition [115,116,117] in the deafferented VNs. The release of BDNF by reactive microglia, acting on its receptor TrkB, was shown to be involved in the downregulation of KCC2 [71,118,119,120]. However, it has been shown that the release of neurotrophic factors such as BDNF is under the control of M2 microglia [37,106,107] which may explain why KCC2 downregulation was not observed in our UVN model following acute LPS treatment. Interestingly, the same enhanced KCC2 expression has also been observed in depression [121] and epilepsy [122] which are both linked with chronic neuroinflammation and elevated levels of pro-inflammatory cytokines [107,123]. This highlights a potential link between an increased pro-inflammatory environment and a potential enhanced neuronal inhibition through KCC2 up-regulation.

#### 4.2.4. Stress Response Is Related to the Inflammatory State of the Deafferented Vestibular Environment following UVN

UVL is known to induce an activation of the hypothalamic-pituitary-adrenal (HPA) axis leading to the release of endogenous corticosteroids (ECs) with anti-inflammatory properties in the deafferented VN [11,18,115]. The HPA axis activation following UVL interacts with vestibular compensation [17] and glucocorticoid receptor (GR) activation is necessary for the expression of the plasticity mechanisms in the deafferented VN [124]. Consistently, we observed an activation of GR following UVN [11]. Following acute LPS-treatment, GR activation kinetics was significantly altered both during the acute and chronic phases after UVN. In particular, we observed a significant downregulation of GR activation following LPS treatment during the acute phase, which was also observed after acute corticotherapy [11]. As systemic administration of LPS is known to activate the HPA axis [51], we might argue that the combination of vestibular injury and acute LPS treatment could lead to corticosteroid overexposure in the deafferented VN, causing GR downregulation [125]. Interestingly, corticosteroid overexposure has been shown to inhibit the M2 microglial phenotype [126] which could promote preferential polarization towards an M1 phenotype as observed in the deafferented VN after acute LPS treatment. In line with our previous results [11], we suggest, here, the existence of a close crosstalk between the stress response and the inflammatory state of the deafferented VNs that might interfere with the expression of the plasticity mechanisms.

### 4.3. Modulation of the Inflammatory State in the Deafferented VNs Interferes with Functional Recovery

Vestibular compensation is defined as the spontaneous and progressive functional recovery following UVL [7]. It depends, among other processes, on the restoration of the electrophysiological balance between ipsi- and contra-lesional VNs, involving the expression of multiple adaptive plasticity mechanisms in the deafferented VNs (for review see [8,9]). We observed that the acute LPS treatment alters neurogliogenesis, KCC2 dynamics, and GR expression in the deafferented VN, all known to play a role in vestibular compensation [52,53,124,127]. Here we used various behavioral methods to assess vestibular, postural, and locomotor functions, especially adapted to vestibular compensation in the rat model of UVN [50,61,66,68].

To evaluate vestibular syndrome intensity, we used a qualitative scale encompassing typical postural and locomotor deficits induced by UVN, whose progressive disappearance over time reflect vestibular compensation [50,59]. Acute LPS-treatment increases vestibular deficits, but only during the acute phase according to this test, which is not consistent with our quantitative and automatized assessments indicating long-term deficits. It should be noted that the use of qualitative scales to assess vestibular syndrome and vestibular compensation has been criticized because of their lack of sensitivity. They do not always match with quantitative assessments of vestibular recovery [74] which are more precise and are performed automatically.

We observed enhanced and long-term deficits following acute LPS treatment regarding the support surface, the rearing time, i.e., time spent on the two hind paws, and posturographic assessments of barycenter stability. These postural parameters are considered markers of postural function [12,61,66]. Particularly, the support surface measured after TLR is thought to reflect the effectiveness of the vestibular function in maintaining postural stability during vestibulospinal reflex [63,64,65]. Taken together, these results show that acute treatment with LPS causes long-term deficits in the recovery of postural function, probably caused by the disruption of plasticity mechanisms in the deafferented vestibular environment.

Interestingly, we observed no real impact of the acute LPS treatment on the lateral weight distribution and locomotor activity, as previously observed following acute anti-inflammatory treatment [11]. This might suggest that the weight increase toward the ipsilesional paws and the increased locomotion during the chronic phase of the vestibular syndrome are not linked to the plasticity expressed within the VNs but are, rather, new compensatory strategies developed following UVN. Indeed, previous works have reported bilateral network restructuration at both subcortical and cortical levels following UVL, proposed to reflect sensorimotor reorganization during vestibular compensation [65,67,128].

### 4.4. Therapeutic Perspectives

In line with our previous study demonstrating that acute anti-inflammatory treatment alters vestibular compensation [11], we show here that modulation of the endogenous inflammatory state following UVN has a deleterious impact on vestibular compensation. Our study highlights that the inflammatory state of the environment following UVN conditions the expression of endogenous plasticity mechanisms and functional recovery. In particular, we show here, for the first time the importance of microglia’s reaction and sequential activation of both M1 and M2 phenotypes for vestibular compensation. Interestingly, we recently demonstrated that sensorimotor rehabilitation [74] or pharmacological treatment with L-thyroxin [129] improves vestibular compensation by promoting microgliogenesis in the deafferented VNs. Microglia therefore appear to be a key and privileged target for pharmacological or rehabilitation therapy to improve vestibular compensation.

While systemic inflammation has been described in vestibular patients [130], the evidence of a central inflammatory process has not been demonstrated so far. Most acute peripheral vestibulopathies (APV) involve peripheral damage to the vestibular system. However, it should be noted that animal models of peripheral vestibular injuries induce a central inflammatory response [10,13,14,15,16]. While further investigations need to be undertaken, inflammation and microglial cells should be considered as therapeutic targets to improve the management of vestibular patients. In particular, aging increases the risks of peripheral and central damage to the vestibular system and about 35% of older adults suffer from vestibular dysfunction (for reviews see [131,132]). Aging is usually associated with poor vestibular compensation both in animals [133,134,135] and humans [136]. Interestingly, the neuroinflammatory response appears to be age-dependent and aging is associated with enhanced microglial reaction [137]. Aging microglia have been shown to promote M1 activation in pathological conditions [138,139]. An imbalance between M1 and M2 microglial activation could therefore occur in aged vestibular patients and enhances impaired compensation, as observed during acute LPS treatment in our UVN rodent model. Although further studies are necessary, pharmacological agents modulating microglial polarization could be useful tools to promote functional recovery.

## Data Availability

Data presented in this study are available upon request from the corresponding author.
